# The Importance of Correlation between Aldosterone and Parathyroid Hormone in Patients with Primary Hyperparathyroidism

**DOI:** 10.1155/2022/3804899

**Published:** 2022-11-07

**Authors:** Branka Milicic Stanic, Branislava Ilincic, Radmila Zeravica, Dragana Milicic Ivanovski, Velibor Cabarkapa, Romana Mijovic

**Affiliations:** ^1^Department of Medicine, Georgetown University Medical Center, 4000 Reservoir Road, NW, Washington, DC 20057, USA; ^2^University of Novi Sad, Faculty of Medicine, Hajduk Veljkova 3, Novi Sad 21000, Serbia; ^3^Center for Laboratory Medicine, Clinical Center of Vojvodina, Hajduk Veljkova 1–9, Novi Sad 21000, Serbia; ^4^Center for Pathophysiology of Breathing and Respiratory Sleep Disorders, The Institute for Pulmonary Diseases of Vojvodina, Put dr Goldmana 4, Sr Kamenica 21204, Serbia

## Abstract

In primary hyperparathyroidism, an increased risk of developing the cardiovascular disease may exist due to increased activity of the renin-angiotensin-aldosterone system. The aim of this study was to evaluate the relationship between parathyroid hormone and aldosterone in patients with primary hyperparathyroidism. The study included 48 patients with primary hyperparathyroidism and 30 healthy subjects who matched age and gender to the study group. This study was conducted at the Center for Laboratory medicine, Clinical center of Vojvodina, Novi Sad, Serbia. In addition to clinical data and laboratory determination of the concentration of total and ionized calcium, phosphorus, measurements of parathyroid hormone, vitamin D, direct renin, and aldosterone were performed by the method of chemiluminescent technology. Compared to the controls, the study group had statistically significantly higher values of aldosterone (*p*=0.028), total calcium (*p*=0.01), ionized calcium (*p*=0.003) and parathyroid hormone (*P* ≤ 0.001) Serum aldosterone and parathyroid hormone levels were correlated positively in patients with primary hyperparathyroidism (*r*=0.509, *p* < 0.05). A statistically significant positive correlation between renin and parathyroid hormone (*r*=0.688, *p* < 0.05) and renin and calcium (*r*=0.673, *p* < 0.05) was determined in hyperparathyroid patients. In multivariate regression analysis, the strongest predictive variable of aldosterone secretion was parathyroid hormone (*p*=0.011). An independent relationship between parathyroid hormone and aldosterone in patients with primary hyperparathyroidism and the correlation between renin and parathyroid hormone as well as with calcium indicate not only the direct but also the indirect associations between parathyroid hormone and aldosterone in primary hyperparathyroidism. These findings may represent another possible model of renin-angiotensin-aldosterone-induced organ damage.

## 1. Introduction

Parathyroid glands synthesize the parathyroid hormone (PTH) which is the primary regulator of calcium homeostasis. After the structure and function of the PTH hormone was clarified, it became clear that PTH also manifests other effects in addition to the action on the bones and kidney, such as the effect on the cardiovascular system [1, 2].

PTH is a peptide hormone secreted by the chief cells of the parathyroid glands [3]. The major physiological regulator of PTH secretion is the concentration of serum ionized calcium (Ca^2+^) [4]. Ca^2+^ is a ligand for the calcium-sensitive receptor (CaSR), presented on the surface of the parathyroid glands, C-cells of the thyroid gland, and also in the kidneys and the brain. Decreased concentration of calcium (Ca^2+^) in the blood will be registered by CaSR receptors and trigger activity in parathyroid cells that leads to the secretion of already synthesized hormone stored in the secretory granules of the main cells of the parathyroid gland [1, 5]. In addition to calcium, other regulators of PTH secretion have been identified such as primarily phosphates and calcitriol but also magnesium, catecholamines, fibroblast growth factor 23 (FGF-23)/klotho system, estrogen, etc. [4].

PTH interacts with type 1 PTH receptor (PTH/PTHrP receptors) located primarily on the plasma membrane of the renal tubules and bone cells-osteoblasts but also in other tissues including fibroblasts, chondrocytes, smooth muscle cells of the blood vessels, cardiomyocytes, adipocytes, and placental trophoblasts [2, 6].

PTH increases the level of calcium by renal tubular reabsorption, bone resorption and promotion of conversion of 25 (OH)D to the active metabolite of vitamin D-1, 25 (OH)_2_D_3_ which further stimulates intestinal Ca absorption [1].

Hyperparathyroidism is a common endocrine disorder and various studies revealed an estimated prevalence of primary hyperparathyroidism (pHPT) in the general population of 0.5% to 1% increasing with age over 2% [7, 8].

Primary hyperparathyroidism is a disease caused by overactive secretion of PTH characterized by hypercalcemia, hypophosphatemia, symptoms of osteoporosis, kidney stones, and numerous neurologic and cardiovascular disorders [9].

There are numerous of evidences that patients with adenoma or parathyroid gland hyperplasia, i.e., primary hyperparathyroidism, have an increased risk of developing a cardiovascular disease [10].

PTH has a positive inotropic effect, and in adult cardiomyocytes, PTH binding activates G-protein signaling, increasing calcium influx into cardiac cells through beta-adrenergic stimulation [11]. The important role of PTH is maintaining normal cardiac contractility by having a positive chronotropic effect in cardiomyocytes [12].

The results of numerous studies have indicated that PTH promotes Ca^2+^ entry into cells, increasing vasoconstriction and causing arterial hypertension [13]. Also, PTH promotes vascular and cardiac remodeling, causes vascular stiffness, and increases cardiac afterload [14]. Cardiovascular morbidity and mortality are increased in both primary and secondary hyperparathyroidism, with a 25–70% prevalence of hypertension in primary hyperparathyroidism, according to different sources [15–17]. There are several predisposing mechanisms that affect the onset of hypertension in primary hyperparathyroidism: PTH leads to increased synthesis of 1, 25 (OH)_2_ vitamin D, which then leads to increased calcium influx into the smooth muscle cells of the blood vessels causing elevated vascular tone and arterial hypertension. PTH has been shown to have a prosclerotic effect on blood vessels which contributes to atherosclerosis of blood vessels and the development of hypertension [18–22].

The renin-angiotensin-aldosterone system (RAAS) plays an important role in the physiological regulation of sodium and potassium balance, intravascular volume, and blood pressure [23].

Aldosterone is a mineralocorticoid that is a major regulator of extracellular fluid volume and potassium metabolism [1]. The biological effects of angiotensin II and aldosterone are mediated through the mineralocorticosteroid angiotensin type 1 and type 2 receptors (ATR1 and ATR2) [24]. Hypersecretion of aldosterone increases sodium reabsorption in the distal tubules, and potassium and hydrogen secretion, leading to hypervolemia, arterial hypertension with imbalance of ionic composition, and acid-base status. Due to increased aldosterone production, numerous adverse cardiovascular, renal, and cerebrovascular effects result from the activation of mineralocorticoid receptors, which are widespread in endothelial cells, smooth muscle cells, cardiomyocytes, parathyroid cells, endothelial progenitor cells, and neutrophils [25]. According to experimental data, aldosterone by a rapid, so-called nongenomic effects, that involve activation of second messenger pathways, in various nonepithelial tissues increases oxidative stress, inflammation, and collagen remodeling leading to endothelial dysfunction, ventricular hypertrophy, cardiac fibrosis, heart failure, and kidney damage and is directly associated with the development of atherosclerosis [26].

It is very important to understand the normal physiological relationship between the RAAS and PTH because it has been previously established that dysregulation of aldosterone as well as PTH plays an important role in the development and progression of cardiovascular disease.

Recent studies revealed that calcium and PTH have been implicated in regulating the RAAS, and the RAAS, in turn, has been implicated in regulating these calcium-regulatory hormones.

Recent observational studies in individuals with primary hyperaldosteronism (PA) suggest that excess aldosterone may result in hyperparathyroidism [18, 27]. However, the interaction between these two hormones and their potential role in target organ damage are less well known.

There are an increasing number of studies indicating a two-way and positive relationship between the RAA system and PTH [28].

### 1.1. Research Objective

The aim of the study was to examine the relationship between circulating serum levels of parathyroid hormone and aldosterone in patients with primary hyperparathyroidism.

## 2. Material and Methods

### 2.1. Respondents and Study Design

This cross-sectional study was conducted in the period from February 2017 to April 2018, in the Clinical Center of Vojvodina, Novi Sad, Serbia.

48 patients who were biochemically diagnosed with primary hyperparathyroidism were included confirmed by positive sestamibi radionuclide scan.

The basic criteria for exclusion of respondents were verified metabolic bone disease, hyperaldosteronism, Cushing's syndrome, pheochromocytoma, type 1 and type 2 diabetes mellitus, autoimmune diseases, previously proven terminal renal failure, and malignant diseases. The control group consisted of 30 healthy subjects with the normal activity of the parathyroid glands and adrenal glands who matched the study group according to age and gender.

After taking anamnestic data and data from the medical documentation, the subjects underwent laboratory analysis at the Center for laboratory medicine, Clinical Center of Vojvodina, as well as scintigraphy of the parathyroid glands at the Center for Nuclear Medicine, Clinical Center of Vojvodina.

All subjects who entered the study had blood drawn from the cubital vein, after a 12-hour fast and after 15 minutes of rest.

### 2.2. Laboratory Analysis

Laboratory diagnostics included the determination of the concentration of N-TACT PTH, direct renin, aldosterone, total and ionized calcium, phosphorus, and vitamin D.

The methodology of analysis performed in the Center for Laboratory Medicine of the Clinical Center of Vojvodina is as follows: Aldosterone, Direct renin, N-TACT PTH, and vitamin D were determined by the method of direct chemiluminescent technology (CLIA) on an automated Liaison XL, DiaSorin system. Aldosterone/renin ratio (ARR) was calculated. Total calcium and phosphorus were determined by photometric colorimetric method on an automated system Advia 1800, Siemens. Ionized calcium was determined by the ion selective electrode method on the AVL 9180 analyzer, Roche Dg.

For all subjects with pPTH, scintigraphy of the parathyroid glands was performed according to the guidelines by the Parathyroid Task Group of the European Association of Nuclear Medicine (99 mmTc-methoxyisobutylisonitrile (MIBI), gamma camera Symbia E, Siemens) [29].

### 2.3. Ethical Statement

The study has been approved by the Ethics committee of the Medical Faculty of Novi Sad, the University of Novi Sad with the corresponding ethical approval code No. 01-39/165/1. Informed consent was obtained for experimentation with human subjects.

### 2.4. Statistical Analyses

The collected data are presented in the tables as mean values and ±SD for parameters that had a normal distribution, and median and lower and upper quartile (25 and 75%) for parameters that did not have a normal distribution, which was examined by Kolmogorov Smirnov test. Comparison of clinical characteristics, including demographic and administrative data (PHPT-related clinical features, medications), was performed with *χ*^2^ tests for categorical variables. For comparison of biochemical parameters, *t*-test for parametric continuous variables, and the Mann–Whitney *U* test for nonparametric continuous variables was used. The correlation between variables was tested by correlation analysis, by calculating the Pearson and Spearman correlation coefficients. The relationship between aldosterone, 25 (OH) vitamin D, and N-TACT PTH was tested by linear regression analysis. A two-tailed*p* < 0.05 was considered significant.

The program STATISTICA 14 (Statsoft Inc, Tulsa, OK, USA) was used for statistical data processing.

## 3. Results

The clinical characteristics of the study and the control group are shown in Table 1.

By testing the statistical significance of the differences between the study and control groups, statistically significant differences were found for all examined parameters except for age (*p*=0.415) and gender (Table 1), renin (*p*=0.17) and aldosterone/renin ratio (*p*=0.88) (Table 2). Compared to the control group, the study group had statistically significantly higher levels of aldosterone (*p*=0.028), total calcium (*p*=0.01), ionized calcium (*p*=0.003) and PTH (*p* ≤ 0.001), while statistically significantly lower levels in the study group were for phosphorus (*p*=0.003) and 25 (OH) D (*p*=0.04) (Table 2).

**Figure 1:**
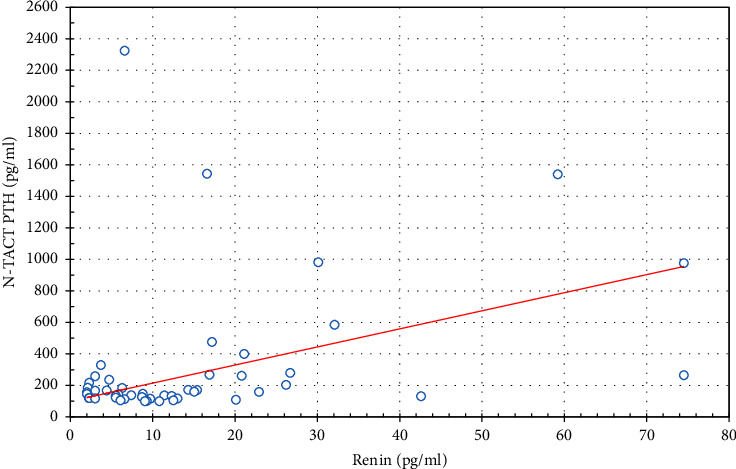
Correlation between PTH and renin in the study group (*r* = 0.688, *p* < 0.05).

**Figure 2:**
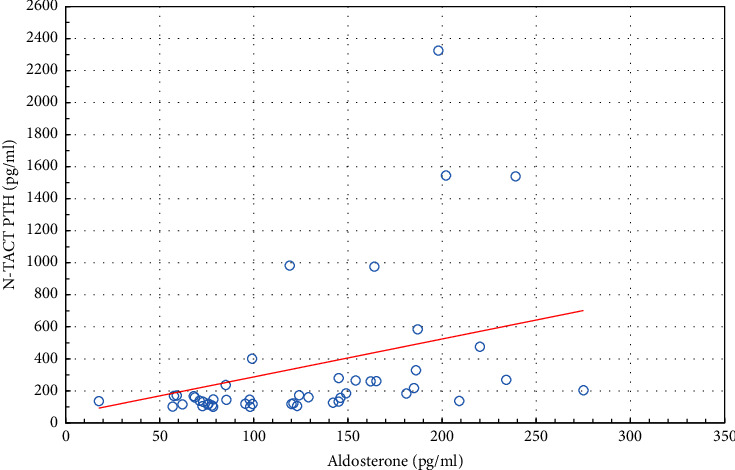
Correlation between PTH and aldosterone in the study group (*r* = 0.509, *p* < 0.05).

In the study group, correlation analysis shows a positive correlation between renin and PTH (*r*=0.688, *p* < 0.05) (Figure 1); renin and calcium (*r*=0.673, *p* < 0.05) and renin and ionized calcium (*r*=0.641, *p* < 0.05). Aldosterone showed a statistically significant positive correlation with PTH (*r*=0.509, *p* < 0.05) (Figure 2) (Table 3).

Correlation analysis in the control group revealed that there was no significant correlation between renin and PTH (*p* > 0.05), calcium (*p* > 0.05) and ionized calcium (*p* > 0.05). Also, aldosterone did not show a positive correlation with PTH (*p* > 0.05), as well as with total and ionized calcium (*p* > 0.05; *p* > 0.05) (Table 3).

No significant correlation was found between 25 (OH)D and other parameters.

In patients with pPTH, linear regression analysis further revealed that PTH levels were significantly associated with aldosterone (*β* = 2.710; *p*=0.011).

## 4. Discussion

In our study, the patients with primary hyperparathyroidism had statistically significantly higher values of PTH (*p* ≤ 0.001), total calcium (*p*=0.01), ionized calcium (*p*=0.003) and aldosterone (*p* = 0.028) compared to the healthy controls, while a statistically significant lower level in the study group was for phosphorus (*p*=0.003) and vitamin D (*p*=0.04). Also, among patients with hyperparathyroidism, a strong positive correlation between aldosterone and PTH was demonstrated (*r* = 0.509, *p* < 0.05), while no correlation between aldosterone and PTH was found among subjects with normal PTH concentration (*p* = −0.285, *p* > 0.05). The most valuable finding in this study was the independent association of aldosterone and PTH (*β* = 2.710; *p* = 0.011).

These data imply that there is a possible association between the PTH and RAAS in patients with primary hyperparathyroidism. Earlier studies indicated that activation of the RAA system is associated with high circulating PTH concentrations. In a cross-sectional study of 192 hypertensive patients, patients with primary aldosteronism had higher plasma PTH concentrations than patients with essential hypertension [30]. The molecular background as a possible link between the various components of PTH and RAAS has not yet been sufficiently studied, but an increasing number of studies suggest that there is a bidirectional association between aldosterone and PTH. It is known that cells of the parathyroid gland possess the mineralocorticoid receptor and the PTH receptor type I is present in the adrenal gland [31]. The effect of PTH on the concentration of aldosterone in the plasma may be direct or indirect, related to the concentration of the renin in the plasma [32]. PTH has been shown to increase aldosterone production from adrenocortical cells and to affect angiotensin II-induced adrenal aldosterone secretion [32, 33], which is in concordance with our data showing a strong positive correlation between PTH and renin as well as with aldosterone. A research team of Mazzocchi et al. documented that both PTH and parathyroid hormone-like peptide-PTHrP stimulate aldosterone secretion in the zona glomerulosa of the adrenal gland by binding to PTH/PTH-rP receptors and activating the adenylate cyclase/protein kinase C cascade as well as phospholipase C/protein kinase C dependent signaling cascade in a dose dependent manner [34]. Although we did not provide experimental data, our finding of that PTH is the strongest predictive variable of aldosterone secretion supports previous study results. Previous results from clinical studies indicate the same direction as our research. In the study by Brown et al., it was observed that patients with the highest PTH values were those who had high serum aldosterone and low renin activity (the “primary aldosteronism” phenotype). On the other hand, patients with the secondary aldosteronism phenotype (high renin activity and high aldosterone) had a PTH level that did not differ from subjects with low or normal aldosterone levels, suggesting that the phenotype of hyperaldosteronism and chronically elevated aldosterone may affect PTH elevation [27]. These results are in concordance with another study in patients with PA that had a significant increase of PTH, pointing on the bidirectional link between zona glomerulosa of the adrenal gland and parathyroid gland [35]. In a study by Brunaud et al., it was shown that aldosterone concentrations in patients with primary hyperparathyroidism before surgery correlated positively with PTH levels in these patients [32]. There are some conflicting studies evaluating the correlation between aldosterone and PTH levels in hypertensive patients, before and after parathyroidectomy, without alteration of the RAAS after surgery [36]. In another study, the authors concluded that patients with high levels of PTH after parathyroidectomy have a more severe disease [37]. Therefore, from future studies evaluating the impact of parathyroidectomy in symptomatic and asymptomatic patients with primary hyperparathyroidism, we will gain more knowledge whether surgery decreased risk of cardiovascular mortality in all patients in the PHPT group.

Our results showed a strong positive correlation between PTH and renin concentration in patients with primary hyperparathyroidism. These data are in concordance with the results of the studies suggesting that PTH may be a direct or indirect stimulator of plasma renin activity. Grant et al., in order to examine the effect of PTH on renin secretion, gave patients an infusion of a parathyroid hormone-like peptide, which resulted in increased aldosterone secretion and tetrahydroaldosterone excretion but also an increase in plasma renin concentration [38]. This direct stimulation of PTH on renin secretion is possible since PTH has a direct effect on juxtaglomerular cells because PTH receptors are detected on isolated glomerular blood vessels [39].

Our study showed a strong positive correlation between total and ionized calcium and renin, but this type of relationship was absent between Ca and aldosterone in patients with hyperparathyroidism. A number of studies have shown that conditions with chronically elevated ionized calcium concentrations or with activation of calcium-sensitive receptors are associated with increased plasma renin activity [31, 40]. These data point to another, indirect mechanism of renin increase caused by chronic hypercalcemia. Also, that supports the theory of indirect stimulation of aldosterone by PTH via renin release. Atchison et al. showed that the chronic hypercalcemia can indirectly reduce the level of aldosterone by weakening the response to angiotensin II [40].

Elevated circulating PTH is considered as an independent risk factor for cardiovascular events and mortality [10, 41]. Fisher et al. documented that PHPT is associated with a higher rate of hypertension [42]. Also, cardiovascular morbidity and mortality have been shown to be increased in patients with elevated plasma aldosterone concentrations [43]. An interplay between PTH and aldosterone can increase cardiovascular risk and can adversely affect the various cardiovascular diseases [44]. In primary hyperparathyroidism, an increased risk of developing the cardiovascular disease may exist due to increased activity of the RAA system. Thus, in patients with hyperparathyroidism, who have in adenomatous altered tissue increase in the expression of type 1 angiotensin II receptors, the concomitant hyperaldosteronism can aggravate arterial-stiffness, endothelial dysfunction, blood pressure elevation, cardiac hypertrophy and cardiovascular disease [11, 12]. In our study, statistically significantly more patients had a hypertension than subjects of matched sex and age in a control group which supports previous data.

On the other hand, in a large retrospective study including normotensive PHPT patients, Castellano et al. did not find a significant correlation between aldosterone and PTH [45]. These results are suggesting that in RAAS activation in PHPT patients, a positive correlation can be found between aldosterone levels and PTH, rather than in a normotensive state.

Taking into account all these data, one can also hypothesized that detecting the level of aldosterone is necessarily in primary hyperparathyroidism in order to spot and treat the disorder in time.

A large number of studies support clinically relevant interactions between parathyroid hormone and aldosterone, as our research has shown. It has been suggested that treatment of primary hyperparathyroidism, may have a positive effect on the cardiovascular system, reducing the activity of PTH and consequently aldosterone [31].

## 5. Conclusion

An independent relationship between parathyroid hormone and aldosterone in patients with primary hyperparathyroidism and the correlation between renin and PTH as well as with calcium indicate not only the direct but also the indirect associations between PTH and aldosterone in primary hyperparathyroidism. These findings may represent another possible mode of RAAS-induced organ damage and should help in finding appropriate diagnostic methods to estimate a cardiovascular risk and an adequate treatment to prevent cardiovascular disease in primary hyperparathyroidism.

## Figures and Tables

**Table 1:** Clinical differences between the study and control group.

Variables	Study group PHPT (*N* = 48)	Control group (*N* = 30)	*p*
Age (years)	66 (52–72)	61 (54–68)	NS
Female (*n*/*N*) (%)	40 (83.3)	24 (80.0)	NS
Kidney stones (*n*/*N*)	12 (25.0)	3 (10.0)	0.001
Osteoporosis (*n*/*N*)	14 (29.2)	4 (13.3)	0.004
Hypertension (*n*/*N*) (%)	28 (58.3)	9 (30.0)	0.028
ACE inhibitors (*n*/*N*) (%)	10 (35.7)	4 (44.4)	NS
Diuretics (*n*/*N*) (%)	9 (32.1)	3 (33.3)	NS
Beta blockers (*n*/*N*) (%)	11 (39.3)	4 (44.4)	NS

Normally distributed variables presented as *x* ± SD and skewed variables by median (interquartile range); categorical variables presented as *n* (%); NS—not significant; PHPT—Primary hyperparathyroidism; ACE—angiotensin converting enzyme.

**Table 2:** Differences in biochemical parameters between the study and control group.

Variables	Study group PHPT (*N* = 48)	Control group (*N* = 30)	*p*
Renin (pg/ml)	9.49 (5.1–18.6)	7.82 (4.5–11.3)	NS
Aldosterone (pg/ml)	126.59 ± 58.07	96.37 ± 58.17	0.028
Aldosterone/renin ratio	11.17 (5.97–22.3)	11.48 (7.96–17.12)	NS
PTH (pg/ml)	159.5 (120.9–263)	74 (65–92.70)	≤0.001
Calcium (mmol/l)	2.72 ± 0.39	2.52 ± 0.17	0.010
Ca^++^ (mmol/l)	1.27 ± 0.19	1.15 ± 0.09	0.003
Phosphorus (mmol/l)	0.82 ± 0.20	0.95 ± 0.18	0.003
25 (OH) D (nmol/l)	48.86 ± 22.54	62.45 ± 23.75	0.040

Normally distributed variables presented as *x* ± SD and skewed variables by median (interquartile range); NS—not significant; PHPT—Primary hyperparathyroidism; Ca^++^—ionized calcium.

**Table 3:** Correlation in the study and the control group for the examined variables.

Study group PHPT (N=48)						

Variables	PTH (pg/ml)		Total calcium (mmol/l)		Ionized calcium (mmol/l)	

	r	p	r	p	r	p

Renin (pg/ml)	0.688	<0.05	0.673	<0.05	0.641	<0.05

Aldosterone (pg/ml)	0.509	<0.05	0.189	>0.05	0.166	>0.05

Control group (N=30)						

Variables	PTH (pg/ml)		Total calcium (mmol/l)		Ionized calcium (mmol/l)	
	r	p	r	p	r	p

Renin (pg/ml)	-0.383	>0.05	-0.060	>0.05	-0.118	>0.05

Aldosterone (pg/ml)	-0.285	>0.05	-0.200	>0.05	-0.313	>0.05

Legend: PTH – Parathyroid hormone; PHPT-Primary hyperparathyroidism

## Data Availability

The data used to support the results of our study are shown within the article.

## Abbreviations

PTH: Parathyroid hormone
Ca^2+^: Ionized calcium
CaSR: Calcium-sensitive receptor
FGF-23: Fibroblast growth factor 23
pHPT: Primary hyperparathyroidism
RAAS: Renin-angiotensin-aldosterone system
ATR1: Angiotensin type 1 receptors
ATR2: Angiotensin type 2 receptors
PA: Primary hyperaldosteronism
ACE: Angiotensin converting enzyme.
